# Selective laser trabeculoplasty versus eye drops for first-line treatment of ocular hypertension and glaucoma (LiGHT): a multicentre randomised controlled trial

**DOI:** 10.1016/S0140-6736(18)32213-X

**Published:** 2019-04-13

**Authors:** Gus Gazzard, Evgenia Konstantakopoulou, David Garway-Heath, Anurag Garg, Victoria Vickerstaff, Rachael Hunter, Gareth Ambler, Catey Bunce, Richard Wormald, Neil Nathwani, Keith Barton, Gary Rubin, Marta Buszewicz, LiGHT Trial Study Group, Gareth Ambler, Keith Barton, Rupert Bourne, David Broadway, Catey Bunce, Marta Buszewicz, Amanda Davis, Anurag Garg, David Garway-Heath, Gus Gazzard, Rachael Hunter, Hari Jayaram, Yuzhen Jiang, Evgenia Konstantakopoulou, Sheng Lim, Joanna Liput, Timothy Manners, Stephen Morris, Neil Nathwani, Gary Rubin, Nicholas Strouthidis, Victoria Vickerstaff, Sarah Wilson, Richard Wormald, Haogang Zhu

**Affiliations:** a NIHR Biomedical Research Centre at Moorfields Eye Hospital NHS Foundation Trust, London, UK; b Institute of Ophthalmology, University College London, London, UK; c Marie Curie Palliative Care Research Department, UCL Division of Psychiatry, University College London, London, UK; d The Research Department of Primary Care and Population Health, University College London, London, UK; e Department of Statistical Science, University College London, London, UK; f School of Population Health and Environmental Sciences, Faculty of Life Sciences and Medicine, King's College London, London, UK; g London School of Hygiene & Tropical Medicine, London, UK

## Abstract

**Background:**

Primary open angle glaucoma and ocular hypertension are habitually treated with eye drops that lower intraocular pressure. Selective laser trabeculoplasty is a safe alternative but is rarely used as first-line treatment. We compared the two.

**Methods:**

In this observer-masked, randomised controlled trial treatment-naive patients with open angle glaucoma or ocular hypertension and no ocular comorbidities were recruited between 2012 and 2014 at six UK hospitals. They were randomly allocated (web-based randomisation) to initial selective laser trabeculoplasty or to eye drops. An objective target intraocular pressure was set according to glaucoma severity. The primary outcome was health-related quality of life (HRQoL) at 3 years (assessed by EQ-5D). Secondary outcomes were cost and cost-effectiveness, disease-specific HRQoL, clinical effectiveness, and safety. Analysis was by intention to treat. This study is registered at controlled-trials.com (ISRCTN32038223).

**Findings:**

Of 718 patients enrolled, 356 were randomised to the selective laser trabeculoplasty and 362 to the eye drops group. 652 (91%) returned the primary outcome questionnaire at 36 months. Average EQ-5D score was 0·89 (SD 0·18) in the selective laser trabeculoplasty group versus 0·90 (SD 0·16) in the eye drops group, with no significant difference (difference 0·01, 95% CI −0·01 to 0·03; p=0·23). At 36 months, 74·2% (95% CI 69·3–78·6) of patients in the selective laser trabeculoplasty group required no drops to maintain intraocular pressure at target. Eyes of patients in the selective laser trabeculoplasty group were within target intracoluar pressure at more visits (93·0%) than in the eye drops group (91·3%), with glaucoma surgery to lower intraocular pressure required in none versus 11 patients. Over 36 months, from an ophthalmology cost perspective, there was a 97% probability of selective laser trabeculoplasty as first treatment being more cost-effective than eye drops first at a willingness to pay of £20 000 per quality-adjusted life-year gained.

**Interpretation:**

Selective laser trabeculoplasty should be offered as a first-line treatment for open angle glaucoma and ocular hypertension, supporting a change in clinical practice.

**Funding:**

National Institute for Health Research, Health and Technology Assessment Programme.

## Introduction

Glaucoma is a progressive multifactorial disease characterised by damage to the optic nerve and progressive visual loss that, if left untreated, can lead to blindness. It is a significant cause of visual morbidity, accounting for falls,^1^ road traffic accidents, loss of independence,^2^ and 12% of blind registrations.^3^ Open angle glaucoma (OAG) is the most common form, with a prevalence of about 2% among adults older than 40 years;^4^ it is strongly associated with elevated intraocular pressure. Lowering intraocular pressure can slow progression of the disease and is the only treatment available.^5^ Raised intraocular pressure without optic nerve damage is termed ocular hypertension, which, in some patients, progresses to open angle glaucoma; lowering intraocular pressure reduces this risk.^6^

The standard first-line treatment for OAG and ocular hypertension is eye drops that lower intraocular pressure, requiring multiple hospital visits for monitoring and treatment adjustment. Long-term and multiple topical medications are associated with multiple ocular and systemic side-effects, poor patient adherence, and are a risk factor for later surgical failure.^7^, ^8^ Selective laser trabeculoplasty reduces intraocular pressure by increasing aqueous outflow through the trabecular meshwork with a single, painless outpatient laser procedure, minimal recovery time, and good safety profile. It was introduced in 1995 and received US FDA approval in 2001, yet is not routinely offered as first-line treatment. Selective laser trabeculoplasty superseded argon laser trabeculoplasty, with fewer adverse events, greater ease of use, and improved repeatability.^9^ The intraocular pressure-lowering effect is comparable to medical treatment and can delay or prevent the need for eye drops, avoiding the associated side-effects. The effect of selective laser trabeculoplasty is not permanent, but it can be repeated. When successful, it reduces the risk of non-adherence, by removing or lessening the need for complex treatment regimes.

**Research in context**

**Evidence before this study**

A Cochrane systematic review published in 2007, highlighted the need for research comparing the clinical efficacy and cost-effectiveness of selective laser trabeculoplasty to eye drops, for lowering intraocular pressure for the treatment of open angle glaucoma or ocular hypertension. We did a literature search in June, 2018. We used the MEDLINE through PubMed using the search terms “selective laser trabeculoplasty”; “SLT”; “laser trabeculoplasty”; and “original research studies”.

Two meta-analyses were published in 2015, showing that 360-degree selective laser trabeculoplasty gives a similar reduction in intraocular pressure to either prostaglandin analogue monotherapy or combination therapy. The studies reviewed had adopted various follow-up periods and a wide range of success criteria. In terms of cost-effectiveness, selective laser trabeculoplasty has been modelled to be cost-effective when compared to medical lowering of intraocular pressure, but no studies used direct measurements of costs. Since the publication of the Cochrane systematic review, the time threshold at which selective laser trabeculoplasty becomes cost-effective against intraocular pressure-lowering drops has been modelled and estimated to be 1–3·3 years, depending on the cost of drops. Selective laser trabeculoplasty has also been predicted to be cost-effective when repeated once within 3 years of initial application compared to monotherapy or multiple drug therapy. The economic data available have been based on the Canadian, US, or Australian health-care systems.

**Added value of this study**

This trial shows that selective laser trabeculoplasty is safe and effective as a first-line treatment for open angle glaucoma and ocular hypertension. Selective laser trabeculoplasty provides superior intraocular pressure stability to drops, at a lower cost and, importantly, it allows almost three quarters of patients (74%) to be successfully controlled without drops for at least 3 years after starting treatment. This is the first trial of a direct comparison between selective laser trabeculoplasty and intraocular pressure-lowering drops in terms of health-related quality of life, clinical, and cost-effectiveness outcomes in a pragmatic hospital setting, guided by a robust treatment escalation protocol to capture realistic clinical management while minimising risk of bias. It also suggests that measurements of generic health-related quality of life have limited discriminatory power in demonstrating treatment effects in glaucoma.

**Implications of all the available evidence**

Selective laser trabeculoplasty is associated with lower cost, good clinical outcomes, with lower symptom scores, and drop-freedom for most patients and should be offered as an alternative to intraocular pressure-lowering drops.

Glaucoma has an adverse effect on health-related quality of life, through progressive loss of field of vision and the inconvenience and side effects of treatments including eye drops and surgery.^10^, ^11^ The treatment of OAG and ocular hypertension also imposes significant financial costs. The cost benefits of selective laser trabeculoplasty have been modelled for a variety of health-care systems,^12^ but direct evidence on its cost-effectiveness as a primary treatment is lacking.

We carried out a multicentre randomised controlled trial to compare eye drops versus selective laser trabeculoplasty as first-line treatment for OAG or ocular hypertension. We also compared the clinical effectiveness and cost-effectiveness of the two approaches. We hypothesised that selective laser trabeculoplasty as a first-line treatment would be associated with better health-related quality of life, less need for topical medication, and lower cost.

## Methods

### Study design and participants

Details of the trial design and baseline characteristics of the Laser in Glaucoma and ocular HyperTension (LiGHT) study are described elsewhere.^13^, ^14^ Consecutive, newly referred patients were identified at six hospitals across the UK (appendix p 1) between Oct 10, 2012, and Oct 27, 2014. Eligible patients had newly diagnosed, untreated OAG or ocular hypertension in one or both eyes, qualified for treatment according to NICE guidelines,^15^ and, for those with OAG, had visual field loss with mean deviation not worse than −12 dB in the better eye or −15 dB in the worse eye and corresponding damage to the optic nerve. Patients were aged 18 years or older, able to read and understand English, had a visual acuity of 6/36 or better in the eyes to be treated, and no previous intraocular surgery, except uncomplicated phacoemulsification at least 1 year before randomisation. Patients were excluded if there were contraindications to selective laser trabeculoplasty (eg, unable to sit at the slit-lamp mounted laser, past history of uveitis, inadequate view of trabecular meshwork), if they were unable to use eye drops, had symptomatic cataract, or were under active treatment for another ophthalmic condition. Patients were monitored for 36 months.

We wished to capture the complexities of normal clinical practice, including any effects that knowledge of treatment might have on patient behaviour, and yet to avoid potential bias arising from clinical decision making when patients and clinicians were not masked to treatment allocation. To achieve this, we used disease severity and pre-treatment intraocular pressure to set objective patient-specific intraocular pressure targets, treatment intensities, and monitoring intervals (adjusted on the basis of intraocular pressure control, disease stability, or adverse reactions). This approach was guided by a defined protocol, using decision support software based on published criteria. Deviations from decision support-recommended interventions were recorded.^14^

The study was conducted in accordance with good clinical practice guidelines and adhered to the tenets of the Declaration of Helsinki. Ethical approval was granted by local boards of each participating centre. All patients provided written informed consent before participation. An independent data and safety monitoring committee was appointed by the independent trial steering committee, to whom adverse events were reported according to standard operating procedures. The protocol is available online.

### Randomisation and masking

We randomised participants using a web-based system (www.sealedenvelope.com) We randomly assigned patients (1:1) to either eye drops or selective laser trabeculoplasty as first-line treatment. We used stratified randomisation, with diagnosis (ocular hypertension *vs* OAG) and treatment centre as stratification factors, with random block sizes (of four, six, or eight). All measurements influencing treatment escalation decisions (visual field, optic disc imaging, and intraocular pressure) were made by observers masked to treatment allocation. Clinicians and patients were not masked to treatment allocation.

### Procedures

Patients with one or both eyes eligible were treated identically. Participants were treated along two treatment pathways depending on their random allocation: either topical medication to lower intraocular pressure (the eye drops group) or primary selective laser trabeculoplasty followed by topical medications as required (the selective laser trabeculoplasty group). We used NICE thresholds^15^ for disease definition (OAG or ocular hypertension) and treatment initiation, incorporated into real-time web-based clinical decision support software, which was based on optic disc analysis using Heidelberg retina tomography (Heidelberg Engineering, Heidelberg, Germany), automated visual field assessment using the Humphrey Field Analyzer Mark II Swedish interactive threshold algorithm standard 24-2 programme (Carl Zeiss Meditec, Dublin, CA, USA), and intraocular pressure measurements (Goldmann applanation tonometry with daily calibration). Disease category and stage were defined at baseline, using preset objective severity criteria from the Canadian Target IOP Workshop^16^ with additional central visual field loss criteria according to Mills and colleagues.^17^

Eye-specific target intraocular pressure and patient follow-up intervals were based on the Canadian Target IOP Workshop^16^ for disease severity stratification (mild, moderate, or severe), with the target intraocular pressure determined from both a percentage reduction (20% or 30% depending on the patient's clinical characteristics) from a single untreated baseline measurement and an absolute threshold. Deterioration of glaucoma (ie, progression) and conversion of ocular hypertension to OAG was derived from the decision support software (based on visual field and Heidelberg retina tomography data) and verified by a consultant ophthalmologist. Objective visual field and optic nerve head imaging criteria using the incorporated Humphrey Glaucoma Progression Analysis software, as used by the Early Manifest Glaucoma Trial,^18^ requiring four consecutive visual field tests, and Heidelberg retina tomography rim area, respectively, defined strong evidence and less strong evidence of deterioration. Strong evidence was defined as “likely progression” according to Glaucoma Progression Analysis or Heidelberg retina tomography rim area greater than 1% per year (p <0·001); less strong evidence was defined as “possible progression” on Glaucoma Progression Analysis or Heidelberg retina tomography rim area greater than 1% per year (p<0·01).^14^

Treatment escalation followed international guidelines of the European Glaucoma Society,^19^ American Academy of Ophthalmology Preferred Practice Pattern,^20^ and South-East Asia Glaucoma Interest Group.^21^ Treatment was escalated when there was either: (1) strong evidence of deterioration irrespective of intraocular pressure, (2) intraocular pressure above the target by more than 4 mm Hg at a single visit, or (3) intraocular pressure above the target by less than 4 mm Hg and less strong evidence of progression. Target intraocular pressure was reduced by 20% if deterioration was identified despite the measured intraocular pressure being at or below target. If the intraocular pressure was above target by less than 4 mm Hg, but with no evidence of deterioration, then the target intraocular pressure was revised to the mean of the previous three visits over which deterioration had not occurred.

Standardisation of laser delivery was achieved by protocol-defined settings and clinical endpoints.^14^ Selective laser trabeculoplasty was delivered to 360° of the trabecular meshwork. 100 non-overlapping shots (25 per quadrant) were used, with the laser energy varied from 0·3 to 1·4 mJ by the clinician, using an appropriate laser gonioscopy lens. One re-treatment with selective laser trabeculoplasty was allowed, provided there had been a reduction in intraocular pressure after the initial treatment; the next escalation was medical therapy. Significant complications of selective laser trabeculoplasty (eg, a spike in intraocular pressure) precluded repetition of selective laser trabeculoplasty.

Drug classes for first, second, or third line treatment were defined by NICE^15^ and European Glaucoma Society^19^ guidance (first line was prostaglandin analogues, second line was β blockers, third or fourth line was topical carbonic anhydrase inhibitors or α agonists). Fixed combination drops were allowed. Systemic carbonic anhydrase inhibitors were only permitted while awaiting surgery. Maximum tolerated medical therapy was defined by the treating clinician as the most intensive combination of drops an individual could reasonably, reliably, and safely use and thus varied between patients. A need for treatment escalation beyond maximum tolerated medical therapy triggered an offer of surgery.

An adverse event was defined as an unfavourable medical occurrence in a patient, not necessarily caused by treatment. Adverse events were classified as serious according to good clinical practice guidelines.^22^ Adverse events were reported according to standard operating procedures and good clinical practice guidelines, and reported annually to the research and ethics committee and the trial sponsor.

### Outcomes

The primary outcome measure was health-related quality of life measured using the EuroQol EQ-5D 5 Levels (EQ-5D-5L) utility scores at 36 months. Utility scores were calculated from patient reported health states using the EQ-5D descriptive system and value set for England.^23^ The secondary outcomes were: glaucoma-specific treatment-related quality of life assessed with the Glaucoma Utility Index (GUI),^24^ patient-reported disease and treatment-related symptoms assessed using the Glaucoma Symptom Scale (GSS);^25^ patient-reported visual function assessed using the Glaucoma Quality of Life-15 questionnaire (GQL-15); health-care resource use, collected from patients' files and from a modified version of the Client Service Receipt Inventory (appendix pp 11–15), clinical effectiveness (proportion of visits at target intraocular pressure, number of treatment escalations), visual function (visual acuity, visual fields), and safety. The EQ-5D, GUI, GSS, GQL-15, and Client Service Receipt Inventory were completed at 6-monthly intervals by postal questionnaire, backed up by telephone or online follow-up.

### Statistical analysis

The statistical analysis plan has been described in detail previously.^26^ We calculated that a sample size of 718 patients was needed to detect a difference of 0·05 in EQ-5D between the two groups using a two sample *t* test at the 5% significance level with 90% power, assuming a common standard deviation of 0·19 and 15% attrition.

The primary outcome was analysed using linear regression with terms for randomisation group, baseline EQ-5D, stratification factors (diagnosis and centre), baseline intraocular pressure, and number of eyes affected at baseline. The unit of analysis was the patient. If a patient had both eyes in the study, baseline severity and intraocular pressure were based on the worse eye, where the worst eye was defined using visual field loss with mean deviation at baseline. Missing values of EQ-5D at 36 months were imputed using values from 30 months, when available. Several sensitivity analyses were performed to verify the results of the primary analysis (appendix p 25). In addition, mixed effects models were used to analyse the EQ-5D measurements recorded at all timepoints to investigate possible changes in treatment effect over the 36 months (using interaction terms between randomisation group and time) and to estimate the average treatment effect over the 36-month follow-up period.

The secondary outcomes were analysed using similar regression methods to those described above. All analyses were performed on an intention-to-treat basis with participants analysed according to the group to which they were allocated. All analyses were performed in Stata (version 14).

For our economic assessment, we calculated quality-adjusted life-years (QALYs) from utility scores over the 36-month period using the baseline and 6-monthly follow-up EQ-5D questionnaires and calculating the area under the curve. Health-care resource use was based on cost using published sources (appendix pp 23–24). The cost of drops for OAG and ocular hypertension based on prescribed medications, using the British National Formulary.^27^ We report cost-effectiveness acceptability curves and the probability that the intervention is cost-effective for a range of values of willingness to pay. Full details of the methodology for the economic evaluation can be found in the appendix (pp 19–25).

This study is registered at controlled-trials.com (ISRCTN32038223).

### Role of the funding source

The funder of the study had no role in study design, data collection, data analysis, data interpretation, or writing of the report. The corresponding author had full access to all the data in the study and had final responsibility for the decision to submit for publication.

## Results

**Figure 1:**
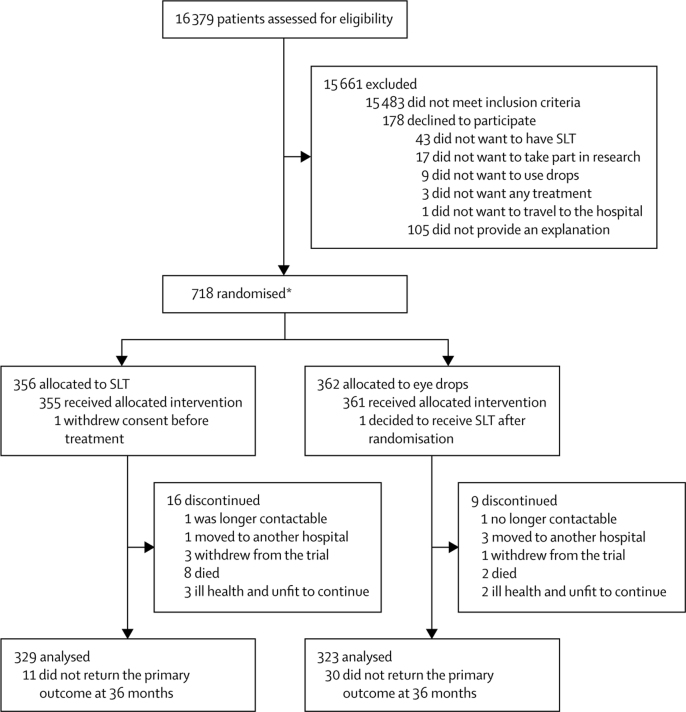
Trial profile SLT=selective laser trabeculoplasty. *Two patients were randomised twice due to computer failure: one was initially randomised to medication but was subsequently randomised to, and received, SLT. The other was initially randomised to SLT but was subsequently randomised to, and received, medication. These patients are included in the figure according to their second randomisations. In addition, four other patients who did not meet the eligibility criteria (and could not receive treatment) were randomised in error. These patients were subsequently removed from the study and are not included in the total randomised.

718 patients (1235 eyes) were randomly assigned: 356 patients (613 eyes) to SLT and 362 patients (622 eyes) to eye drops (figure 1). One patient (two eyes) allocated to selective laser trabeculoplasty withdrew consent before treatment. Two patients were assigned twice because of a computer error, where the initial randomisation was not visible. Subsequently, a second randomisation for these patients was carried out; one patient was initially randomised to eye drops but was subsequently randomised to, and received, selective laser trabeculoplasty. The second patient was initially randomised to selective laser trabeculoplasty but was later randomised to, and received, eye drops. Four patients who did not meet the eligibility criteria were randomised in error and were subsequently removed from the study. Data for the primary outcome were available for 652 (91%) of 718 patients at 36 months (329 [92%] of 356 in the selective laser trabeculoplasty group and 323 [89%] of 362 in the eye drops group) and were included in the intention-to-treat analysis (with imputation used for missingness; figure 1). There were 30 protocol violations or deviations without an effect on safety or clinical outcomes (four non-eligible patients were randomised, one received eye drops despite randomisation to selective laser trabeculoplasty, and one had a third selective laser trabeculoplasty at the consultant's discretion; there were 17 incorrect appointment intervals, five incorrect visual field software versions, one Heidelberg retina tomography was not done, and the decision support software was inappropriately run once).

Baseline patient and eye characteristics were similar in the treatment groups (Table 1, Table 2); 555 patients had OAG (in at least one eye) and 163 had ocular hypertension. In 517 (72·0%) of 718 patients both eyes were eligible, in 96 patients (13·4%) only the right was eligible, and in 105 patients (14·6%) only the left. The two treatment groups had similar average EQ-5D, GUI, and GQL-15 scores at baseline (table 3). The eye drops group had slightly higher average GSS scores at baseline compared to the selective laser trabeculoplasty group (table 3).

**Table 1:** Baseline patient characteristics

		**Eye drops (n=362)**	**SLT (n=356)**
Centre			
	Moorfields Eye Hospital	187 (51·7%)	187 (52·5%)
	Huntingdon Hospital	41 (11·3%)	41 (11·5%)
	Guy's and St Thomas' Hospital	55 (15·2%)	51 (14·3%)
	Queen's University Belfast	15 (4·1%)	15 (4·2%)
	Norfolk and Norwich University Hospital	46 (12·7%)	43 (12·1%)
	York Hospital	18 (5.0%)	19 (5·3%)
Mean age (years, SD)		62·7 (11·6%)	63·4 (12·0%)
Sex			
	Male	197 (54·4%)	200 (56·2%)
	Female	165 (45·6%)	156 (43·8%)
Ethnicity*			
	Asian	28 (7·7%)	23 (6·5%)
	Black	69 (19·1%)	77 (21·6%)
	White	258 (71·3%)	243 (68·3%)
	Other	7 (1·9%)	13 (3·7%)
Diagnosis			
	OAG	282 (77·9%)	273 (76·7%)
	OHT	80 (22·1%)	83 (23·3%)
Other health conditions			
	Asthma	45 (12·4%)	48 (13·5%)
	Hypertension	119 (32·9%)	132 (37·1%)
	Diabetes	40 (11·1%)	42 (11·8%)
	Angina	11 (3·0%)	10 (2·8%)
	Cardiac arrhythmia	20 (5·5%)	17 (4·8%)
Medication			
	Statins	92 (25·4%)	104 (29·2%)
	Systemic β blockers	12 (3·3%)	22 (6·2%)
	Calcium channel blocker	60 (16·6%)	56 (15·7%)
	ACE inhibitors	43 (11·9%)	57 (16·0%)
	Corticosteroids	20 (5·5%)	22 (6·2%)
Family history of glaucoma†		107 (29·6%)	107 (30·1%)
Highest education achieved			
	Degree or equivalent (≥16 years of education completed)	106 (29·3%)	110 (30·9%)
	Higher education (≥15 years)	39 (10·8%)	55 (15·5%)
	A-level or equivalent (13 years)	49 (13·5%)	39 (11.0%)
	GCSEs (11 years)	84 (23·2%)	71 (19·9%)
	Other qualifications (≥11 years)	30 (8·3%)	29 (8·2%)
	No qualifications (11 years)	54 (14·9%)	52 (14·6%)

SLT=selective laser trabeculoplasty. OAG=primary open angle glaucoma. OHT=ocular hypertension. ACE=angiotensin-converting enzyme inhibitor.

* Self-defined ethnicity as per National Health Service categories: Asian ethnicity refers to Indian, Pakistani, Bangladeshi, and any other south Asian background; black ethnicity refers to Caribbean, African, and any other black background; other ethnicity refers to Chinese and any other ethnic group.

† In a first degree relative.

**Table 2:** Baseline ocular characteristics

		**Number of eyes with data (patients)**	**Eye drops (n=622 eyes; 362 patients)**	**SLT (n=613 eyes; 356 patients)**
Diagnosis		1235 (718)	··	··
	Ocular hypertension	··	185 (29·7%)	195 (31·8%)
	Mild OAG	··	325 (52·3%)	311 (50·7%)
	Moderate OAG	··	77 (12·4%)	67 (10·9%)
	Severe OAG	··	35 (5·6%)	40 (6·5%)
Refractive error (spherical D)		1225 (713)	−0·2 (2·7)	−0·3 (3·2)
Visual acuity		1235 (718)	0·1 (0·1)	0·1 (0·2)
Visual field mean deviation (dB)		1233 (717)	−3·0 (3·6)	−3·0 (3·4)
HRT rim area (mm^2^)		1128 (656)	1·1 (0·4)	1·2 (0·4)
Intraocular pressure (mm Hg)		1233 (717)	24·4 (5·0)	24·5 (5·2)
CCT (μm)		1229 (715)	551·6 (36·2)	550·7 (38·1)
Pseudo-exfoliation		1233 (717)	12 (1·9%)	5 (0·8%)
Pseudophakia		1233 (717)	33 (5·3%)	39 (6·4%)

Data are n (%) or mean (SD). SLT=selective laser trabeculoplasty. OAG=primary open angle glaucoma. HRT=Heidelberg retina tomograph. CCT=central corneal thickness.

**Table 3:** Baseline questionnaire scores

		**Eye drops (n=362)**	**SLT (n=355)**
EQ-5D*		0·92 (0·13)	0·91 (0·13)
Glaucoma Utility Index*†		0·89 (0·11)	0·89 (0·12)
Glaucoma Symptom Scale†‡		83·3 (16·6)	81·4 (17·2)
	Symptom subscale	81·2 (19·4)	79·1 (20·1)
	Function subscale	86·4 (17·3)	84·8 (17·8)
Glaucoma Quality of Life-15§		18·7 (5·6)	18·9 (6·6)
	Central subscale	2·5 (1.0)	2·5 (1.0)
	Peripheral subscale	8·4 (2·9)	8·5 (3·4)
	Dark subscale	7·9 (2·8)	7·9 (3.0)
	Outdoor subscale	1·1 (0·4)	1·1 (0·4)

Data are mean (SD). SLT=selective laser trabeculoplasty. EQ-5D=EuroQol EQ-5D.

* n=716.

† Higher scores indicate better health-related quality of life.

‡ n=710.

§ Higher scores indicate worse health-related quality of life.

**Figure 2:**
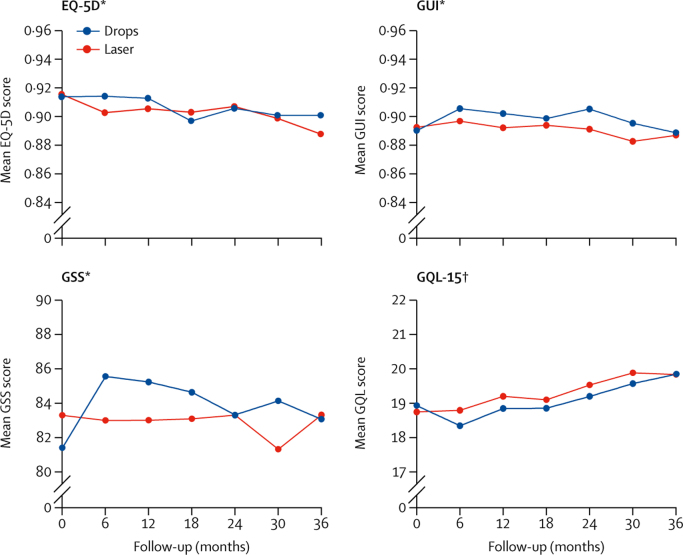
Mean EQ-5D, GUI, GSS, and GQL-15 scores at each time point, across 36 months Time-point ‘0’ refers to pre-treatment. EQ-5D=EuroQol 5 Dimensions 5 Levels. GUI=Glaucoma Utility Index. GSS=Glaucoma Symptom Scale. GQL-15=Glaucoma Quality of Life-15. *Higher scores indicate better health-related quality of life. †Higher scores indicate worse health-related quality of life.

At 36 months, the eye drops group had an average EQ-5D score of 0·90 (SD 0·16), compared with 0·89 (SD 0·18) in the selective laser trabeculoplasty group, with no significant difference between the two treatments (adjusted mean difference [selective laser trabeculoplasty–eye drops] 0·01, 95% CI −0·01 to 0·03, p=0·23; table 4, figure 2); the results were confirmed in sensitivity analyses (data not shown).

**Table 4:** Primary and secondary analysis of health-related quality-of-life questionnaires

		**Eye drops**		**SLT**		Adjusted mean difference*(95% CI)	**p value**
		n	Mean (SD)	n	Mean (SD)		
**Analysis at 36 months**							
EQ-5D		336	0·89 (0.18)	337	0·90 (0.16)	0·01 (−0·01 to 0·03)	0·230
GUI		299	0·89 (0·13)	303	0·89 (0·13)	0·01 (−0·01 to 0·03)	··
GSS		281	83·3 (17·3)	294	83·1 (17·7)	1·6 (−0·8 to 4·0)	··
GQL-15		297	19·8 (7·8)	304	19·8 (7·2)	−0·4 (−1·3 to 0·6)	··
QALY		263	2·70 (0·42)	261	2·74 (0·37)	0·025 (−0·02 to 0·07)	0·289
QALY (discounted)		263	2·62 (0·41)	261	2·65 (0·36)	0·024 (−0·02 to 0·07)	0·286
**Repeated measures analysis**							
EQ-5D							
	Baseline	362	0·92 (0·13)	355	0·91 (0·13)	··	··
	6 months	332	0·90 (0·15)	330	0·91 (0·13)	0·01 (−0·01 to 0·03)	··
	12 months	327	0·91 (0·14)	327	0·91 (0·14)	0·01 (−0·01 to 0·02)	··
	18 months	329	0·90 (0·16)	325	0·90 (0·16)	0·00 (−0·02 to 0·02)	··
	24 months	326	0·91 (0·14)	326	0·91 (0·14)	0·00 (−0·02 to 0·02)	··
	30 months	320	0·90 (0·15)	317	0·90 (0·15)	0·00 (−0·01 to 0·02)	··
	36 months	323	0·89 (0·18)	329	0·90 (0·16)	0·02 (−0·00 to 0·03)	··
GUI							
	Baseline	361	0·89 (0·11)	355	0·89 (0·12)	··	··
	6 months	330	0·90 (0·11)	329	0·91 (0·10)	0·01 (−0·00 to 0·03)	··
	12 months	315	0·89 (0·12)	320	0·91 (0·11)	0·01 (−0·00 to 0·03)	··
	18 months	305	0·89 (0·12)	303	0·90 (0·13)	0·01 (−0·01 to 0·02)	··
	24 months	298	0·89 (0·12)	305	0·90 (0·11)	0·02 (0·00 to 0·03)	··
	30 months	299	0·88 (0·12)	291	0·89 (0·12)	0·02 (0·00 to 0·03)	··
	36 months	300	0·89 (0·13)	303	0·89 (0·13)	0·01 (−0·01 to 0·02)	··
GSS							
	Baseline	357	83·3 (16·6)	353	81·4 (17·2)	··	··
	6 months	321	83·0 (16·3)	320	85·6 (14·9)	4·0 (2·0 to 6.0)	··
	12 months	310	83·0 (17·6)	309	85·2 (15·4)	2·9 (0·8 to 4·9)	··
	18 months	295	83·1 (16·8)	294	84·6 (15·8)	2·8 (0·7 to 4·8)	··
	24 months	287	83·3 (16·4)	290	83·3 (16·3)	1·4 (−0·7 to 3·5)	··
	30 months	288	81·3 (17·6)	276	84·1 (16·7)	3·5 (1·5 to 5·6)	··
	36 months	282	83·3 (17·3)	296	83·1 (17·7)	2·2 (0·1 to 4·2)	··
GQL-15							
	Baseline	361	18·7 (5·6)	355	18·9 (6·6)	··	··
	6 months	323	18·8 (5·6)	324	18·3 (5·4)	−0·8 (−1·6 to 0·0)	··
	12 months	314	19·2 (7·2)	318	18·8 (6·6)	−0·5 (−1·4 to 0·3)	··
	18 months	302	19·1 (6·4)	298	18·9 (6·5)	−0·6 (−1·4 to 0·2)	··
	24 months	289	19·5 (7·3)	298	19·2 (6·7)	−0·5 (−1·3 to 0·4)	··
	30 months	293	19·9 (7·1)	287	19·6 (7·9)	−0·3 (−1·1 to 0·5)	··
	36 months	298	19·8 (7·8)	304	19·8 (7·2)	−0·4 (−1·2 to 0·4)	··

SLT=selective laser trabeculoplasty. EQ-5D=EuroQol EQ-5D (higher scores represent a better quality of life). GUI=Glaucoma Utility Index (higher scores represent a higher quality of life). GSS=Glaucoma Symptom Scale (higher scores represent better outcomes). GQL-15=Glaucoma Quality of Life-15 (higher scores represent poorer glaucoma quality of life). QALY=quality-adjusted life-years.

* (SLT eye drops); adjusted for baseline score, severity, centre, baseline intraocular pressure, and number of eyes affected at baseline.

Taking into account the outcome data from all timepoints across 36 months, the two treatment arms had similar EQ-5D scores at 36 months (adjusted mean difference 0·02, 95% CI −0·00 to 0·03); and when using exact times of questionnaire returns (0·01, −0·01 to 0·02). The average GUI score at 36 months in the selective laser trabeculoplasty group was 0·89 (SD 0·13) compared with 0·89 (SD 0·13) for the eye drops group (adjusted mean difference 0·01, 95% CI −0·01 to 0·03). Mean GQL-15 scores were also similar between the two groups (19·8 for selective laser trabeculoplasty and 19·8 eye drops, adjusted mean difference −0·4, 95% CI −0·6 to 1·3). For the GSS the selective laser trabeculoplasty group had a mean score of 83·3 (SD 17·3) at 36 months, compared with 83·1 (SD 17·7) for the eye drops group (adjusted mean difference 1·6, 95% CI −0·8 to 4·0). Repeated measures analysis showed worse GSS scores for the eye drops group at five out of six timepoints over 36 months (table 4, figure 2). Secondary outcomes (GUI, GSS, GQL-15) generally suggested better health-related quality-of-life outcomes for the selective laser trabeculoplasty group (figure 2).

At 36 months, 536 (87·7%) of 611 eyes in 314 patients in the selective laser trabeculoplasty group and 536 eyes (86·2%) of 622 eyes in 312 patients in the eye drops group were available for analysis of clinical outcomes (table 5). The groups had similar endpoint visual acuity, intraocular pressure, and visual field loss mean deviation (table 5).

**Table 5:** Measurements of pathway effectiveness and visual function

				**Eye-drops group**	**SLT group**
**Treatment intensity**					
Total number of SLT treatments at 36 months				6*	770
Number of SLT treatments per eye					
	One			6 (1·0%)	453 (74·1%)
	Two			0 (0%)	157 (25·7%)
	Three†			0 (0%)	1 (0·2%)
Number of medications per eye at target IOP at 36 months‡					
	No medication			16 (3·0%)	419 (78·2%)
	One			346 (64·6%)	64 (12·0%)
	Two			99 (18·5%)	21 (3·9%)
	Three			35 (6·5%)	4 (0·8%)
	Four			3 (0·6%)	1 (0·2%)
Eyes not at target at 36 months				37 (7·0%)	27 (5·0%)
**Control of disease**					
Visits at target (cumulative)				91·3%	93·0%
Eyes at target IOP at 36 months				499 (93·1%)	509 (95·0%)
	OHT			127 (92·0%)	151 (95·6%)
	Mild OAG			261 (94·6%)	259 (96·3%)
	Moderate OAG			69 (94·5%)	55 (96·5%)
	Severe OAG			42 (85·7%)	44 (84·6%)
Treatment escalations§				348	299
Disease progression during the trial				36 (5·8%)	23 (3·8%)
	From OHT to OAG¶			3	2
	OAG progression			33	21
			Algorithm defined VF progression	27	18
			Algorithm defined optic disc progression	3	2
			Algorithm defined VF and disc progression	3	1
Ocular surgeries during the trial					
	Phacoemulsification			25	13
	Trabeculectomy			11	0
	Trabeculectomy revision			7 (5 eyes)	0
**Clinical endpoints at 36 months**					
Visual acuity (LogMAR)				0·08 (0·17)	0·07 (0·18)
	OHT			0·08 (0·19)	0·02 (0·15)
	Mild OAG			0·06 (0·15)	0·08 (0·17)
	Moderate OAG			0·12 (0·16)	0·11 (0·24)
	Severe OAG			0·16 (0·23)	0·15 (0·18)
IOP (mm Hg)				16·3 (3·87)	16·6 (3·62)
	OHT			18·7 (3·73)	18·2 (3·73)
	Mild OAG			15·7 (3·45)	16·4 (3·17)
	Moderate OAG			14·7 (3·49)	14·4 (3·07)
	Severe OAG			15·5 (4·17)	15·5 (4·16)
VF MD (dB)				−3·21 (3·76)	−3·19 (3·92)
	OHT			−0·94 (1·92)	−1·05 (1·98)
	Mild OAG			−2·14 (1·95)	−1·99 (1·93)
	Moderate OAG			−7·21 (1·92)	−7·96 (2·04)
	Severe OAG			−10·50 (5·01)	−10·24 (4·93)
**Clinic visits**					
Total number of clinic visits				2907	3441
Total number of clinic visits excluding 2-week IOP check				2907	2976

Data are n (%) unless otherwise stated. SLT=selective laser trabeculoplasty. IOP=intraocular pressure. OHT=ocular hypertension. OAG=primary open angle glaucoma. VF=visual field. MD=mean deviation.

* Three patients (six eyes) in the eye drops group wanted SLT at treatment escalation.

† Protocol deviation.

‡ Includes eyes that had undergone trabeculectomy.

§ Escalations initiated by the algorithm and the clinicians.

¶ Conversion of OHT to OAG required a sign of progression derived from decision support software and verification by a consultant ophthalmologist.

Overall 509 (95%) of 536 eyes treated with selective laser trabeculoplasty were at target intraocular pressure at 36 months. Target intraocular pressure was achieved without intraocular pressure medication in 419 (78·2%) of 536 eyes treated in the selective laser trabeculoplasty group (table 5); of these 321 (76·6%) required only one treatment. 233 of the patients in the selective laser trabeculoplasty group (74·2%, 95% CI 69·3%–78·6%) were drop-free at 36 months. 499 (93·1%) of the 526 eyes treated in the eye drops group were at target intraocular pressure at 36 months, and 346 (64·6%) were using a single medication. Over 36 months, for 93·0% visits (according to decision support software) patients in the selective laser trabeculoplasty group were at target intraocular pressure compared with 91·3% in the eye drops group (table 5).

More treatment escalations took place in the selective laser trabeculoplasty group (n=348) than in the eye drops group (n=299). 36 eyes in the eye drops group showed algorithm-confirmed disease deterioration (three eyes converted from ocular hypertension to OAG and in 33 eyes OAG progressed) compared with 23 eyes in the selective laser trabeculoplasty group (two eyes converted from ocular hypertension to OAG and in 21 eyes OAG worsened). 25 cataract extractions were carried out in the eye drops group compared with 13 in the selective laser trabeculoplasty group. 11 eyes (1·8%) required surgery to lower intraocular pressure (trabeculectomy) in the eye drops group (five eyes had uncontrollable intraocular pressure, four eyes had uncontrollable intraocular pressure and visual field loss progression, one eye had visual field loss progression, and one eye had uncontrollable intraocular pressure, visual field loss progression, and disc progression) compared with none in the selective laser trabeculoplasty group.

There were no sight-threatening complications of selective laser trabeculoplasty (table 6). Cases of reactivation of herpes simplex keratitis (one in each treatment group) and uveitis (two in the selective laser trabeculoplasty group and one in the eye drops group) were similar. In six eyes of six patients, the intraocular pressure rose on the day of laser treatment by more than 5 mm Hg, but only one eye required treatment. There were more ophthalmic-drop-related adverse events reported by patients in the eye drops group (150 aesthetic side effects or ocular allergic reactions reported by 73 patients) compared to the selective laser trabeculoplasty arm (30 events reported by 20 patients). 122 (34·4%) of 355 patients in the selective laser trabeculoplasty group reported transient discomfort, blurred vision, photophobia, and hyperaemia. Adverse events, including variations in number of laser shots able to be delivered, were reported for 14 patients during selective laser trabeculoplasty. Systemic adverse events were similar overall between the two treatment groups (table 6). Drop-related systemic adverse events were reported more often and by more patients in the eye drops group (148 events reported by 52 [14·4%] of 361 patients compared with 87 events reported by 23 (6·5%) of 355 patients in the selective laser trabeculoplasty group). Pulmonary problems and cardiac events were few and balanced between the two groups. Serious adverse events were also similar between the two groups: 95 in 68 patients in the eye drops group and 107 in 64 patients in the selective laser trabeculoplasty group.

**Table 6:** Adverse events

			**Eye drop group (n=362)**		**SLT (n=356)**		**Total (n=718)**	
			Number of events	Number of patients (%)	Number of events	Number of patients (%)	Number of events	Number of patients (%)
Adverse events			1196	260 (71·8%)	906	261 (73·3%)	2096	521 (72·6%)
	Ocular							
		Aesthetic side effects of medication*	117	56 (15·5%)	12	7 (2·0%)	129	63 (8·8%)
		Ophthalmic allergic reactions†	33	17 (4·7%)	18	13 (3·7%)	51	30 (4·2%)
		Reactivation of herpes simplex keratitis	1	1 (0·3%)	1	1 (0·3%)	2	2 (0·3%)
		Uveitis	1	1 (0·3%)	2	2 (0·6%)	3	3 (0·4%)
		Other‡	744	221	459	186	1203	407
	SLT-related ocular							
		Inflammation after SLT	0	0 (0%)	1	1 (0·3%)	1	1 (0·1%)
		IOP spike after SLT§	0	0	6	6 (1.7%)	6	6 (0·8%)
		Other transient events¶	2	1 (0·3%)	171	122 (34·4%)	173	123 (17·2%)
		Patients with an adverse event during SLT procedure||	··	0	··	14 (3·9%)	··	14 (1·9%)
	Systemic**							
		Pulmonary problems††	23	14 (3·9%)	24	12 (3·4%)	47	26 (3·6%)
		Cardiac events	6	5 (1·4%)	8	5 (1·4%)	14	10 (1·4%)
		Drug-related events‡‡	148	52 (14·4%)	87	23 (6·5%)	235	75 (10·5%)
		Other§§	121	82 (22·7%)	117	78 (22%)	238	160 (22·3%)
Serious adverse events			95	68 (18·8%)	107	64 (18·0%)	202	132 (18·4%)
		Ocular¶¶	7	6 (1·7%)	10	8 (2·2%)	17	14 (1·9%)
		Pulmonary problems||||	3	3 (0·8%)	2	2 (0·5%)	5	5 (0·7%)
		Cerebrovascular accidents	1	1 (0·3%)	2	2 (0·5%)	3	3 (0·4%)
		Cardiac events	7	7 (1·9%)	9	8 (2·2%)	16	15 (2·1%)
		Cancer	9	8 (2·2%)	15	13 (3·6%)	24	21 (2·9%)
		Death	2	2 (0·5%)	8	8 (2·2%)	10	10 (1·4%)
		Other systemic	66	50 (15·3%)	61	43 (12·1%)	127	93 (13%)

SLT=selective laser trabeculoplasty. IOP=intraocular pressure.

* Includes excessive lash growth, peri-ocular pigmentation, change in iris colour.

† Includes peri-ocular skin rash.

‡ Includes ocular irritation, discomfort, dry eye, retinal haemorrhages, vision changes, flashes, floaters, conjunctivitis, blepharitis, vascular occlusions, diabetic retinopathy, macular pathology.

§ IOP spike defined as >5 mm Hg; only one eye received treatment.

¶ Includes discomfort, transient blurred vision, transient photophobia, hyperaemia.

|| Includes discomfort, variation in the number of laser shots, angle visualisation issues.

** Not requiring hospital admission.

†† Asthma, shortness of breath, reduced exercise tolerance.

‡‡ includes impotence, depression, somnolence or tiredness, nightmares, taste disturbance, generalised skin rash.

§§ Unrelated events, such as headaches, pain, falls.

¶¶ Includes central retinal artery occlusion, choroidal neovascularisation, epiretinal membrane, angle closure, anterior chamber surgery, corneal pathologies, trauma, and any treatment required for these pathologies.

|||| Requiring hospital admission.

The cost of selective laser trabeculoplasty over the duration of the trial was an additional £205 (95% CI 196 to 213) in the selective laser trabeculoplasty group. Over the 36 months of the trial, drops for OAG and ocular hypertension cost an additional £465 (95% CI 440 to 491) for patients assigned to the eye drops group. The average cost per patient for ocular surgery over 36 months was significantly less for the selective laser trabeculoplasty group compared with the eye drops group (unadjusted difference −£134, SE 43; 95% CI −218 to −50; p=0·002) and for all ophthalmology costs including selective laser trabeculoplasty and drops (unadjusted difference −£451, SE 66; 95% CI −580 to −322; p<0·001). Selective laser trabeculoplasty as first-line treatment resulted in more QALYs than eye drops first (table 4, but the difference was not significant p=0·286), for a lower cost and so no incremental cost-effectiveness ratio is reported. Imputing missing EQ-5D-5L utilities using multiple imputation and accounting for the correlation between costs and QALYs, using seemingly unrelated regression, selective laser trabeculoplasty as first-line treatment costs £458 less than eye drops first with 95% of bootstrap iterations falling between −£585 and −£345 (for specialist eye-related costs) and has a mean incremental QALY of 0·011, with 95% bootstrap iterations falling between −0·024 and 0·050. Over 36 months, discounted and adjusted, at a £20 000 and £30 000 willingness to pay for a QALY gained, there is a 97% and 93% probability that selective laser trabeculoplasty first is more cost-effective than eye drops first when only ophthalmology costs are included, and a 68% chance for both £20 000 and £30 000 when community and non-eye related costs are added (appendix). A greater total number of clinic visits in the selective laser trabeculoplasty group than in the eye drops group (3441 *vs* 2907) was due to an additional protocol-mandated intraocular pressure check 2 weeks after laser (465 visits), at which no complications affecting management were detected and which is no longer part of routine clinical practice.

## Discussion

In this multicentre randomised controlled trial we compared initial treatment of OAG or ocular hypertension using selective laser trabeculoplasty followed by medication, if required, against the standard medication for lowering intraocular pressure alone. We demonstrated that the treatment pathway with initial selective laser trabeculoplasty is cost-effective with no significant difference in health-related quality of life and clinical outcomes, and lower cost compared with the conventional treatment pathway, where medication is used from the outset.

Our treat-to-target design tailored treatment intensity to disease severity and the treatment response. In the eye drops group, 36 patients (5·8%) had disease progression compared with 23 (3·8%) of patients in the selective laser trabeculoplasty group, 74% of whom remained drop-free at 3 years. The laser first approach provided better control of intraocular pressure over the course of 36 months, with more visits at target intraocular pressure compared with eye drops, less intense drop treatment, and with no glaucoma surgeries. This difference may be because control of intraocular pressure after laser relies on patient adherence with treatment; indeed, one report^28^ found patients had drops to lower intraocular pressure available only 69% of the time, and concordance has been reported to range between 76% and 86%.^28^ Selective laser trabeculoplasty has also been proposed to provide better diurnal intraocular pressure stability, from its continuous effect on the trabecular meshwork, in contrast to the necessarily episodic administration of medication.^29^ By 36 months, patients in the selective laser trabeculoplasty group had lower rates of disease deterioration compared with those in the eye drops group) and that 11 eyes required surgery to lower intraocular pressure in the eye drops group, but none in the selective laser trabeculoplasty group.

Primary selective laser trabeculoplasty gave drop-free control intraocular pressure for at least 36 months to 74·2% of patients (95% CI 69·3%–78·6%), substantially higher than in previous studies^30^, ^31^, ^32^, ^33^ with less stringent success criteria, where selective laser trabeculoplasty was used as both a primary and adjunctive treatment. Prior treatment and more severe disease has been suggested to reduce the degree to which selective laser trabeculoplasty lowers intraocular pressure,^34^ possibly explaining our positive results in treatment-naive patients. This is the first study that reports disease control without topical medication, provided by primary selective laser trabeculoplasty, and does so with realistic but stringent targets for intraocular pressure and objective escalation criteria. Pre-trial patient and public involvement activities with glaucoma patients identified drop-free disease control as the most desired outcome. 90% of patients in a focus group felt being drop-free even in one eye only would be of benefit. Concerns about drop use, particularly associated with challenges from cognitive impairment and failing grip strength, were rated a priority by patients in the James Lind Alliance survey of sight loss research questions.^35^

We also demonstrate a greater safety of selective laser trabeculoplasty than previously reported, with low rates of selective laser trabeculoplasty-related adverse events. There were no systemic adverse events as a result of selective laser trabeculoplasty; only one patient in which selective laser trabeculoplasty caused a spike in intraocular pressure required treatment, out of 776 selective laser trabeculoplasties, compared to reported rates up to 28·8%.^31^ This is possibly due to treatment at an earlier stage of disease. The intraocular pressure check conventionally done 2 weeks after selective laser trabeculoplasty did not change management for any of the patients and consequently appears unnecessary. Abandonment of this routine check could further increase the cost-effectiveness of selective laser trabeculoplasty. There was a lower rate of cataract surgery in the selective laser trabeculoplasty group, supporting existing evidence that eye drops to lower intraocular pressure are associated with a greater incidence of nuclear cataract and earlier need for surgical removal.^18^

Use of selective laser trabeculoplasty as the first-line treatment resulted in a significant reduction in the cost of surgery and medication for ocular hypertension and OAG, with an overall cost saving to the NHS of £451 per patient in specialist ophthalmology costs; for every patient given selective laser trabeculoplasty first instead of eye drops the cost savings are greater than the cost of selective laser trabeculoplasty for two additional patients, or equal to the cost of five additional ophthalmology specialist appointments. At a £20 000 willingness to pay for a QALY gained there is a 97% probability that selective laser trabeculoplasty is a cost-effective treatment for OAG and ocular hypertension. Resource use for these costs was collected from patient files and trial monitoring data and hence is likely to be complete, with limited bias as a result of loss to follow-up or missing data. Including non-eye related health-care costs, the average cost per patient for selective laser trabeculoplasty as first treatment remained less than that for eye drops as first treatment, but the differences between the two groups were not significant, with the wide confidence intervals resulting in a 68% probability that selective laser trabeculoplasty first is cost-effective compared with eye drops. Non-ocular health-care cost data are, however, based on self-reported health-care resource use and may be unreliable or incomplete. Expensive systemic adverse events unrelated to ocular hypertension or OAG, such as cancer or cardiac events, may have also skewed the cost results. Previous economic assessments have attempted to estimate the relative costs of selective laser trabeculoplasty using economic modelling or estimates of the treatment costs, instead of a direct cost assessment.^36^ Compared to monotherapy or multiple drug therapy and allowing for repetition of selective laser trabeculoplasty within 2–3 years, cost-savings have been predicted at 6 years for a Canadian health-care system.^12^ The present study was done in an NHS setting, following pragmatic clinical approaches for the treatment of OAG and ocular hypertension and indicates that selective laser trabeculoplasty is cost effective over a 3-year period.

These findings have important implications for patients and health-care systems. Patients are concerned about the use of drops to lower intraocular pressure and widespread uptake of selective laser trabeculoplasty as first line treatment would lead to a drop-free interval of at least 36 months for almost three quarters of patients, while providing savings for the NHS. Where necessary additional selective laser trabeculoplasty (documented to be effective) could increase the duration of this drop-free window. Patients with OAG have an average 9–13 years life expectancy from diagnosis,^37^ and so a 3 year or longer drop-free period might confer significant benefits to their remaining quality of life. The requirement for intense medical or surgical regimes might be deferred or completely averted by selective laser trabeculoplasty with potentially improved surgical success rates and still lower cost.^8^

This study mirrored pragmatic clinical practice by tailoring treatment to the patient. Individual intraocular pressure targets were based on pre-treatment intraocular pressure and disease severity, and adapted both to treatment response and disease progression. Consequently, our findings on disease progression, achievement of target intraocular pressure, and cost are highly relevant to normal clinical practice. The unique treat-to-target design with computerised decision-supported treatment interventions and follow-up intervals captured the complexity of real-life clinical decision making and yet allowed impartial, objective, and unbiased decisions based on clinical observations. An unmasked design was essential to capture any potential effects (adverse or positive) from the patients' perception of their treatments, as may occur in clinical reality. This is because an important benefit of selective laser trabeculoplasty is a drop-free treatment window; a study design requiring a treatment arm with placebo drops would have prevented assessment of benefits attributable to drop freedom. We also sought to capture the impact of treatment on subsequent medication-taking behaviours and concordance. Similarly, treating clinicians had to be unmasked to be able to choose appropriate treatment escalations. Our results are widely generalisable, as we included patients with ocular hypertension and both low-pressure and high-pressure OAG, from a range of backgrounds and ethnic origins. There are also important implications for resource-poor health-care settings, where access to medication is a major barrier to glaucoma treatment. Adequate drop-free control of intraocular pressure for years is a promising treatment approach for regions of Africa where glaucoma prevalence is high. Longer follow-up, already underway, will permit us to answer further questions regarding the effect of prior selective laser trabeculoplasty on later medication-taking behaviour, treatment intensity, and longitudinal health-related quality of life.

Our primary outcome of health-related quality of life, assessed using the EQ-5D questionnaire, a generic tool eliciting utility values in multiple settings, was required by our funder as a requirement of NICE cost-utility analyses. Recently, the sensitivity of EQ-5D in ophthalmology has been questioned and particularly so for glaucoma, which can be asymptomatic, even at levels sufficient to make driving unsafe.^38^ While potentially blinding over longer periods, only small changes in vision occur in the duration of a clinical trial. The above average health-related quality of life at baseline, the weak sensitivity of the EQ-5D to detect glaucoma-specific effects on health-related quality of life,^39^ and relatively short duration of LiGHT compared to the time for disease progression may have contributed to the lack of superiority of selective laser trabeculoplasty with respect to EQ-5D.

Since glaucoma-specific instruments better capture differences in glaucoma severity than does the effect of treatment side effects on health-related quality of life, the lack of a significant difference in the GUI and GQL-15 might also be expected. Differences in health-related quality of life would arise from differences in drop usage, due to inconvenience or side-effects. The GSS evaluates a visual and an ocular comfort related domain, and incorporates treatment side-effect-related measures. Better GSS scores for the selective laser trabeculoplasty group could represent differences that arise from drop usage, but potentially reflect differences in baseline scores between the different treatment groups.

Our data support a change in practice; however, clinicians will need to consider patients' perceptions of the necessity of monitoring visits, in the absence of daily medication. Primary selective laser trabeculoplasty is a cost-effective alternative to drops that can be offered to patients with OAG or ocular hypertension needing treatment to lower intraocular pressure.
