# Department of Error

**DOI:** 10.1016/S0140-6736(19)31503-X

**Published:** 2019-07-06

*Gazzard G, Konstantakopoulou E, Garway-Heath D, et al. Selective laser trabeculoplasty versus eye drops for first-line treatment of ocular hypertension and glaucoma (LiGHT): a multicentre randomised controlled trial.* Lancet *2019; **393:** 1505–16*—In this Article, the data for the laser trabeculoplasty group and the eye drops group were incorrectly switched in several places. The third paragraph of the Results section should have begun: “At 36 months, the selective laser trabeculoplasty group had an average EQ-5D score of 0·90 (SD 0·16), compared with 0·89 (SD 0·18) in the eye drops group…”. The seventh paragraph of the Results section should have begun: “More treatment escalations took place in the eye drops group (n=348) than in the selective laser trabeculoplasty group (n=299)”. In figure 2, the red line should indicate the patient group receiving drops and the blue line should indicate the patient group receiving laser treatment. These corrections have been made to the online version as of July 4, 2019.

