# Supplementary material for: Selective laser trabeculoplasty versus eye drops for first-line treatment of ocular hypertension and glaucoma (LiGHT): a multicentre randomised controlled trial
Source: Lancet. 2019 Apr 13;393(10180):1505–16. doi: 10.1016/S0140-6736(18)32213-X (PMC6495367; doi:10.1016/S0140-6736(18)32213-X)
Supplement: Supplementary appendix [file mmc1.pdf]

THE LANCET

Supplementary appendix

This appendix formed part of the original submission and has been peer reviewed. We post it as supplied by the authors.

Supplement to: Gazzard G, Konstantakopoulou E, Garway-Heath D, et al. Selective laser trabeculoplasty versus eye drops for first-line treatment of ocular hypertension and glaucoma (LiGHT): a multicentre randomised controlled trial. *Lancet* 2019; published online March 9. http://dx.doi.org/10.1016/S0140-6736(18)32213-X.

Supplementary Online Material – 1

Participating Centres in the UK

Participating centre	Local Principal Investigator	Screening start date	Screening end date
**Moorfields Eye Hospital (MEH) City Road**	Mr G Gazzard	October 2012	October 2014
**MEH at St' George's University Hospital**	Mr G Gazzard<br>Mr N Strouthidis		
**MEH at Northwick Park Hospital**	Mr H Jayaram		
**Royal Victoria Hospital Belfast**	Ms SJ Wilson	November 2013	October 2014
**Guys and St Thomas' Hospital**	Mr KS Lim	July 2013	October 2014
**Hinchingbrooke Hospital**	Prof R Bourne	July 2013	October 2014
**Norfolk and Norwich University Hospital**	Mr DC Broadway	July 2013	October 2014
**York Hospital**	Mr T Manners	November 2013	October 2014

Supplementary Online Material – 2

LiGHT Trial- Laser in Glaucoma and Ocular Hypertension

QUESTIONNAIRE BOOKLET

1 **EuroQol questionnaire** We are asking you the following questions to see how you rate your own health. For each of the following questions please place a tick in the box that closest describes your state of health **TODAY**.

1 **Please indicate your level of mobility** *(please tick **one** box)*

- ☐ I have no problems in walking about
- ☐ I have slight problems in walking about
- ☐ I have moderate problems in walking about
- ☐ I have severe problems in walking about
- ☐ I am unable to walk about

2 **Please indicate your level of self-care** (*please tick **one** box)*

- ☐ I have no problems washing or dressing myself
- ☐ I have slight problems washing or dressing myself
- ☐ I have moderate problems washing or dressing myself
- ☐ I have severe problems washing or dressing myself
- ☐ I am unable to wash or dress myself

3 **Please indicate your ability to perform your usual activities e.g. work, study, housework, family or leisure activities** (*please tick **one** box)*

- ☐ I have no problems doing my usual activities
- ☐ I have slight problems doing my usual activities
- ☐ I have moderate problems doing my usual activities
- ☐ I have severe problems doing my usual activities
- ☐ I am unable to do my usual activities

4 **Please indicate your level of pain** (*please tick **one** box)*

- ☐ I have no pain or discomfort
- ☐ I have slight pain or discomfort
- ☐ I have moderate pain or discomfort
- ☐ I have severe pain or discomfort
- ☐ I have extreme pain or discomfort

5 **Please indicate your level of anxiety or depression** (*please tick **one** box)*

- ☐ I am not anxious or depressed

- ☐ I am slightly anxious or depressed
- ☐ I am moderately anxious or depressed
- ☐ I am severely anxious or depressed
- ☐ I am extremely anxious or depressed

6 We would like to know how good or bad your health is **TODAY**.

- This scale is numbered from 0 to 100.
- 100 means the best health you can imagine.
  0 means the worst health you can imagine.
- Mark an X on the scale to indicate how your health is TODAY.
- Now, please write the number you marked on the scale in the box below.

YOUR HEALTH TODAY = ☐

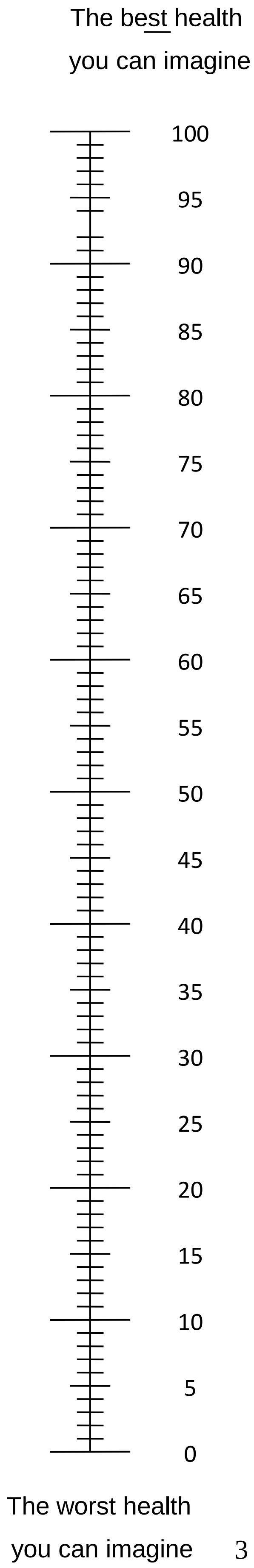

2 Glaucoma Utility Index

"*Tick one* box, for each of the categories 1-6, which best describes any difficulties you have had **in the last month** with your eyes or vision, wearing your usual glasses or contact lenses. Under each category there is an example, to help you answer these questions."

	Level of difficulty			
**Domains**	**No difficulty**	**Some difficulty**	**Quite a lot of difficulty**	**Severe difficulty**
**1. Central and Near Vision**<br>For example difficulties with reading, writing, watching TV, reading dials on clocks?	☐	☐	☐	☐
**2.Lighting and glare**<br>For example difficulties with adjusting from light to dark and vice-versa, bright lights may dazzle, difficulties seeing in dim light?	☐	☐	☐	☐
**3. Mobility**<br>For example difficulties with crossing roads, walking along busy pavements, tripping into low objects e.g. pushchairs?	☐	☐	☐	☐
**4. Activities of daily living**<br>For example difficulties in seeing adequately to do domestic, DIY or self-care tasks around the home?	☐	☐	☐	☐
**5. Eye discomfort**<br>For example difficulties with gritty, sore, tired eyes?	☐	☐	☐	☐
**6. Other effects**<br>For example fatigue, shortness of breath, dry mouth, bitter taste etc?	☐	☐	☐	☐

3 Glaucoma Symptom Scale

Have you experienced any of the following problems with your eyes **in the last 4 weeks**? (Please *tick* a box below for each symptom). If your answer is '*yes*' please tick how bothersome it is.

1 Burning, Smarting, Stinging

No ☐, Go to next question **Yes** ☐, How bothersome is it?

Very ☐
Somewhat ☐
A little ☐
Not at all ☐

2 Tearing ("Watering Eyes")

No ☐, Go to next question **Yes** ☐, How bothersome is it?

Very ☐
Somewhat ☐
A little ☐
Not at all ☐

3 Dryness

No ☐, Go to next question **Yes** ☐, How bothersome is it?

Very ☐
Somewhat ☐
A little ☐
Not at all ☐

Glaucoma Symptom Scale continued …..

4 Itching

No ☐, Go to next question **Yes** ☐, How bothersome is it?

**Very**	☐
**Somewhat**	☐
**A little**	☐
**Not at all**	☐

5 Soreness, Tiredness

No ☐, Go to next question **Yes** ☐, How bothersome is it?

**Very**	☐
**Somewhat**	☐
**A little**	☐
**Not at all**	☐

6 Blurry/dim vision

No ☐, Go to next question **Yes** ☐, How bothersome is it?

**Very**	☐
**Somewhat**	☐
**A little**	☐
**Not at all**	☐

Glaucoma Symptom Scale continued …..

7 Feeling of something in your eye

No ☐, Go to next question **Yes** ☐, How bothersome is it?

**Very**	☐
**Somewhat**	☐
**A little**	☐
**Not at all**	☐

8 Hard to see in daylight

No ☐, Go to next question **Yes** ☐, How bothersome is it?

**Very**	☐
**Somewhat**	☐
**A little**	☐
**Not at all**	☐

9 Hard to see in dark places

No ☐, Go to next question **Yes** ☐, How bothersome is it?

**Very**	☐
**Somewhat**	☐
**A little**	☐
**Not at all**	☐

Glaucoma Symptom Scale continued …..

10 Halos around lights

No ☐ **Yes** ☐, How bothersome is it?

**Very**	☐
**Somewhat**	☐
**A little**	☐
**Not at all**	☐

4. The Glaucoma Quality of Life-15 Questionnaire

Please *Tick one* box, for each of the categories 1-15, which best describes any difficulties you have had in **the last month**, even with your usual glasses or contact lenses.

Activities	None	A little bit of difficulty	Some difficulty	Quite a lot difficulty	Severe difficulty	Do not perform for non visual reasons
1. Reading newspapers	☐	☐	☐	☐	☐	☐
2. Walking after dark	☐	☐	☐	☐	☐	☐
3. Seeing at night	☐	☐	☐	☐	☐	☐
4. Walking on uneven ground	☐	☐	☐	☐	☐	☐
5. Adjusting to bright lights	☐	☐	☐	☐	☐	☐
6. Adjusting to dim lights	☐	☐	☐	☐	☐	☐
7. Going from light to dark room or vice versa	☐	☐	☐	☐	☐	☐
8. Tripping over objects	☐	☐	☐	☐	☐	☐
9. Seeing objects coming from the side	☐	☐	☐	☐	☐	☐

10. Crossing the road	☐	☐	☐	☐	☐	☐
11. Walking up steps/stairs	☐	☐	☐	☐	☐	☐
12. Bumping into objects	☐	☐	☐	☐	☐	☐
13. Judging distance of foot to step/curb	☐	☐	☐	☐	☐	☐
14. Finding dropped objects	☐	☐	☐	☐	☐	☐
15.Recognizing faces	☐	☐	☐	☐	☐	☐

5. The following questions will help Moorfields Eye Hospital collect information about healthcare costs.

1 In the last 6 months have you been in contact with any of the health care services below? If yes please write the number of times below.

**Eye related services**

1A Opticians

**No**	**Yes**	**Number of times**
☐	☐	………………………

1B Hospital eye clinic

**No**	**Yes**	**Number of times**
☐	☐	……………………..

2 **in the last 6 months** have you been admitted to any of the below services? If *'yes'* please enter the number of admissions & total number of nights stay in hospital.

Specialist/Acute hospital services

**2A Overnight in-patient stay**			
**No**, Go to question 2B ☐	**Yes** ☐		
2A1 Planned in-patient admission		**Number of admissions**	**Total number of nights**
**No** ☐	**Yes** ☐	No. = ☐	No. = ☐
2A2 Emergency in-patient admission			
**No** ☐	**Yes** ☐	No. = ☐	No. = ☐
2A3 Intensive care/high dependency unit			
**No** ☐	**Yes** ☐	No. = ☐	No. = ☐
**2B Day patient procedure/test**		**Total number of contacts in the last 6 months**	
**No** ☐	**Yes** ☐	No. = ☐	No. = ☐
**2C Outpatient appointment**			
**No** ☐	**Yes** ☐	No. = ☐	No. = ☐
**2D Accident and Emergency attendance**			
**No,** Go to question 3 ☐	**Yes** ☐		
		Total number of contacts **in the last 6 months**	

2D1 And admitted to hospital

No ☐ **Yes** ☐ No. = ☐ No. = ☐

2D2 Not admitted to hospital

No ☐ **Yes** ☐

3. In the last 6 months, have you used any of the services below? If *'yes'* please enter the number of times you have visited the services below for eye related and non eye related conditions

General Practitioner (GP) and community services

3A GP Contacts

No, Go to question 3B ☐ **Yes** ☐ Total number of contacts in the last **6 months**

		**Eye Related**	**Not eye related**
3A1 In the practice			
**No** ☐	**Yes** ☐	No. = ☐	No. = ☐
3A2 Telephone consultation			
**No** ☐	**Yes** ☐	No. = ☐	No. = ☐
3A3 Home visit			
**No** ☐	**Yes** ☐	No. = ☐	No. = ☐

3B GP Practice Nurse

**No** ☐	**Yes** ☐	No. = ☐	No. = ☐

3C Social Worker

No ☐ **Yes** ☐ No. = ☐ No. = ☐

3D Home care worker

No ☐ **Yes** ☐ No. = ☐ No. = ☐

3E Other Community services

Please give details:___________________ No. = ☐ No. = ☐

4 **In the last 6 months**, have you taken any medication?

No ☐ **Yes** ☐

If yes, what medications have you been taking. Please list below all medications taken (tablets, pills, capsules and all medicines) in the last 6 months

MEDICATION				
**DRUG NAME**	**DURATION OF USE**		**DOSAGE**	
Trade name OR medical name OR name on packet	Please TICK if CONTINOUS	If NOT CONTINOUS please state NO. OF DAYS TAKEN FOR	DOSE TAKEN	NO. TAKEN PER DAY
E.g. Metformin	✓		500mg	2
E.g. Paracetamol		3 days	500mg	3

5. Do you have private health insurance?

Private Health Insurance

No ☐ **Yes** ☐

If *'yes'* please **list below** the health care services you accessed **in the last 6 months** through your private health insurance:

6 Concordance / Compliance

1 **Over the last month,** what percentage or proportion of your eye drops do you think you took correctly? Please write in the boxes below or mark *X* on the scale.

Not applicable ☐

Percentage (%) or proportion taken correctly ☐

%	Proportion
100%	All
75 %	¾
50%	½

2 Please read the below statement and *tick* the box which you agree with.

"I'm the sort of person who follows doctors' orders exactly."

**Statement**	**Please tick one box**
Strongly agree	☐
Somewhat agree	☐
Neither agree nor disagree	☐
Somewhat disagree	☐
Strongly disagree	☐

Supplementary Online Material - 3

Sensitivity analyses for LiGHT

The following sensitivity analyses were performed to verify results of the primary analysis:

1) Estimation of the treatment effect at 36 months using the exact dates of questionnaire completion
2) The analysis was adjusted for variables associated with missingness (age, highest education attainment, employment, and diabetic status)
3) Multiple imputation was used to impute missing outcome data
4) The mapping algorithm was used to calculate the EQ-5D scores
5) The analysis was restricted to those patients whose outcome data were recorded within 3 months (either side) of 36 months
6) Robust standard errors were calculated using the bias corrected and accelerated bootstrap
7) Beta regression was used instead of linear regression.

The results of the sensitivity analysis are presented in the following table:

Approach	Adjusted mean difference* (Laser – drops)	95% CI
**1. Using exact dates**	0.007	(-0.009, 0.022)
**2. Adjusted for variables associated with missingness**	0.011	(-0.008, 0 .030)
**3. Multiple imputation**	0.016	(-0.004, 0.035)
**4. Mapping algorithm**	0.019	(-0.005, 0.042)
**5. Data recorded within 3 months of 36 months**	0.012	(-0.007, 0.031)
**6. Robust standard errors**	0.012	(-0.007, 0.031)
**7. Beta regression**	0.002	(-0.010, 0.013)

* Mean difference is adjusted for baseline score, severity, centre, baseline IOP, and number of eyes affected at baseline.

Supplementary Online Material - 4

Economic Evaluation of LIGHT: trial based cost-utility analysis

1. Aim

The aim of the economic evaluation is to calculate the mean incremental cost per quality adjusted life year (QALY) gained of Laser-1st compared to Medicine-1st. Health and social care costs and QALYs was calculated for the within-trial period (3-years)

2. Outputs

- Mean total patient level QALYs by trial arm
- Mean cost per patient of laser treatment in the Laser-1st arm
- Mean cost per patient of Medicine treatment for glaucoma by trial arm
- Mean cost per patient of surgery by trial arm.
- Mean total health care cost per patient over 3 years by trial arm
- Mean increment cost per QALY of Laser-1st compared to Medicine-1st and 95% confidence intervals
- Cost-effectiveness plane
- Cost-effectiveness acceptability curve

3. Methods

3.1. QALY

Mean patient level QALYs by trial arm are calculated as the area under the curve using patient level responses to EQ-5D-5L at each follow-up time-point (Hunter et al 2015) and the formula by Devlin et al (2017). Patients that died were imputed as zero from the date of death until the end of the trial. We assumed a straight line from the last follow-up time point until death. As the EQ-5D-5L is the primary outcome for the trial, mean patient responses at each follow-up time point are reported in Table 4 of the main paper as part of the repeated measures analysis. The mean incremental difference in QALYs is calculated using ordinary least squares (OLS) regression and including covariates for randomisation group, baseline EQ-5D-5L values, randomisation factors (severity and centre), baseline intra-ocular pressure (IOP), and number of eyes affected at baseline. Further detail on the calculation of each of these factors can be found in the Methods section of the main trial paper.

QALYs are discounted from 12 months to 3 years at an annual rate of 3.5% (NICE 2013). 95% confidence intervals are calculated using bootstrapping, bias corrected and 5,000 replications given that we assume that the data is not normally distributed. Although there was a high rate of return for the EQ-5D-5L at 36 months (91%) data was missing for each time point which meant only 73% of patients had complete data across all time-points for calculation of QALYs. Multiple imputation using chained equations was used to impute the data for 35 datasets including age, highest education attainment, employment, and diabetic status included as variables identified of being predictive of missingness.

3.2. Cost of SLT

The cost of Selective laser trabeculoplasty (SLT) has been calculated using bottom-up micro-costing based on data collected from sites. Sites reported cost of the machine, maintenance costs, how sessions were run (dedicated sessions for SLT or as part of a normal session), the grade and number of staff for each session and the number of patients treated per session and per year. Staff wages and overheads were taken from the Personal Social Services Resource Unit (PSSRU 2018). The cost per patient of the machine is based on an annuitized formula (Drummond et al 2015) accounting for the number of patients seen in a “typical” site per year and assuming a lifetime for the laser of 10 years. The number of SLTs per patient is reported in the main trial paper.

3.3. Cost of Medicine for Open Angle Glaucoma (OAG) and Ocular Hypertension (OHT)

We report the mean cost of Medicine by trial arm over 3 years. Prescription of Medicine, including drug name, dose, number of eyes, number of Medicine per eye and frequency, was collected as part of trial monitoring processes. Each prescription was costed using the British National Formulary (BNF 2018) to calculate the cost per bottle. This was divided by the number of Medicine per bottle to calculate the cost per day. To calculate the number of days per medication it was assumed that patients would take the medication from the day of prescription until the next medication change. The mean total cost per patient is then the cost per day of the prescribed Medicine multiplied by the number of days the medication is prescribed.

3.4. Total ophthalmology related costs

In addition to Medicine and laser, information was collected from patient files on ocular surgery and planned and unplanned specialist ophthalmologist visits. These included a two-week IOP check as part of trial process: this check though would not occur if the service was rolled out and hence this IOP check has been removed from the primary analysis. Descriptive statistics for ophthalmology resource use are reported in the main trial paper in Table 5. Ocular surgery and ophthalmologist outpatient appointments were costed using NHS Reference costs (2018). We report the mean cost per patient at 3 years for each type of ophthalmology cost and total costs, discounted at an annual rate of 3.5% (NICE 2013) by trial arm. 95% bias corrected confidence intervals are calculated using bootstrapping and 5,000 replications. Given data was taken from patient files it's not possible to identify missing data: it is assumed that if patients do not have an appointment or surgery reported it is because none occurred. As a result the ITT is based on all patients assuming that the appointment data collected is correct.

3.5. Other health care costs

Health care resource use including optician, community health care and acute health care contacts was collected from a modified version of the client services receipt inventory (CSRI Beecham et al 19952) at baseline, 6 months, 12 months, 18 months, 24 months, 30 months and 36 months asking about resource use in the past 6 months and for eye related versus not eye related. Patient information on inpatient stays and day cases was checked against serious adverse events (SAE) data. SAEs not reported in the CSRI were included in the total inpatient cost. Resource use was costed using unit costs from PSSRU except for optometrist visits (Violato et al 2016), heart bypass surgery (NHS Reference Costs 2018) and cancer deaths (Georghiou and Bardsley 2014) (Table SM1). Mean costs by trial arm at each time point are by ocular and non-ocular related costs, over 3 years.

Resource use	Unit cost (per contact)	Source
**Trabeculectomy**	1436	NHS Reference costs[1]
**Ophthalmology appointments**	91	NHS Reference costs[1]
**Optometrist visit**	52	Violato et al[2]
**Planned inpatient stay**	3903	PSSRU[3]
**Unplanned inpatient stay – short duration (<7 days)**	628	PSSRU[3]
**Unplanned inpatient stay – long duration (7 days and greater)**	2953	PSSRU[3]
**A&E attendance – and admitted**	221	NHS Reference costs[1]
**A&E attendance – not admitted**	128	NHS Reference costs[1]
**Outpatient attendance**	137	PSSRU[3]
**GP contact – in practice**	31	PSSRU[3]
**GP contact – telephone**	24	PSSRU[3]
**GP contact – at home**	80	PSSRU[3]
**GP practice nurse**	36	PSSRU[3]
**Social care**	59	PSSRU[3]
**Home care**	26	PSSRU[3]
**Other community contacts**	57	NHS Reference costs[1]
**Cancer death**	6129	Georghiou T, Bardsley M.[4]

Table SM1: Health care unit costs used in the cost-effectiveness analysis. All costs are reported in 2016/2017 British Pounds

3.6. Total health and social care costs

The cost components included in the analysis are the cost of SLT, OAG medication and other health care costs. We report the mean cost per patient in addition to an adjusted cost, adjusting for baseline service use using regression analysis. Mean costs are based on a complete case analysis, with only optician and CSRI resource use excluding inpatient stays missing and an analysis imputing for missing CSRI data using chained equations has been included. The mean incremental difference in costs is calculated using OLS regression and includes covariates for randomisation group, baseline EQ-5D-5L values, randomisation factors (severity and centre), baseline intra-ocular pressure (IOP), and number of eyes affected at baseline. We used bias corrected bootstrapping to calculate 95% confidence intervals given that we assume that the data is not normally distributed. All costs are reported in 2016/2017 Great British Pounds (GBP).

3.7. Incremental cost-effectiveness ratio (ICER)

The incremental cost-effectiveness ratio (ICER) is defined the mean incremental cost of Laser-1st compared to Medicine-1st divided by the mean incremental QALYs of laser compared to Medicine. The mean incremental differences are adjusted for baseline values, randomisation factors (severity and centre), baseline intra-ocular pressure (IOP), and number of eyes affected at baseline. To account for the correlation between costs and QALYs seemingly unrelated regression (SUR) was used to calculate the numerator and denominator of the ICER. ICERs are reported for total costs, as defined in 3.6 above and ophthalmology only costs as defined in 3.4 above. Costs and QALYs from 12 months until 36 months are discounted at an annual rate of 3.5% (NICE 2013). The final results for total costs and QALYs are based on data imputed using chained equations for QALYs and CSRI and using the missing at random methodology described in Leurent et al (2018) for calculating CEACs using bootstrapping and multiple imputation for 200 draws of each of the 35 imputed datasets for 7,000 replications in total.

3.8. Cost-effectiveness acceptability curve (CEAC)

A CEAC is reported using the bootstrap imputed data (200 draws of each of the 35 imputed datasets for 7,000 replications in total) for a range of values of willingness to pay for a QALY gained. We report the probability

that Laser-1st is cost-effective compared to Medicine-1st at a willingness to pay for a QALY gained of £20,000 and £30,000 for (a) total costs; (b) ophthalmology only costs.

3.9. Secondary analyses

The following secondary analyses will be conducted:

1) For the primary analysis SLT was costed using micro-costing. Some assumptions of the micro-costing, for example the number of patients per site per year, or how sessions are run, may have an impact on the total cost. As a result we will examine the impact of modifying the assumptions on the total cost per patient of SLT and hence the ICER. The cost of SLT as estimated from NHS Reference Costs will also be used in an analysis.

2) In the primary analysis we have removed the cost of the 2-week IOP check given that this is unlikely to occur in practice. One could hypothesise that patients obtained some minor benefit from this check and hence its costs could be included in the analysis. A secondary analysis including the 2-week IOP check has been included.

3) QALYs will be calculated using utility scores generated from the GUI (Burr et al 2007) and the same methodology for calculating QALYs as above. The results will be combined with costs as above to report the mean incremental cost per QALY gained of Laser-1st compared to Medicine-1st using the GUI.

4. Results

4.1. QALYS

Descriptive statistics for the EQ-5D-5L are reported in Table 4 of the main paper.

In the complete case analysis, the Laser-1st arm has a mean of 2.63 adjusted and discounted QALYs across 3 years (n=261 95% CI 2.60 to 2.66), with 2.61 QALYs in the Medicine-1st arm (n=263 95% CI 2.57 to 2.64) with an adjusted difference of 0.025 (95% CI -0.020 to 0.070 p=0.277). In the multiple imputation analysis there is an adjusted difference of 0.014 (SE 0.220 95% CI -0.029 to 0.057 p=0.526).

4.2. Cost of SLT

There were a range of different models of delivering SLT across the different sites. Although all sites had a dedicated laser session, this was usually mixed in with patients needing other types of laser, not just SLT. The procedure was performed by ophthalmologists of a range of grades, covering Registrar through to Consultant. Supporting staff may be a health care assistant or a lower grade nurse. Sessions tended to last 4 hours with sites quoting between 5 and 8 patients per session. Depending on the number of sessions sites may see between 350 and 200 patients a year for use of the laser (the laser can be used for things other than just SLT).

At a cost of £38,995 for the machine, and an annual maintenance cost of £6395, the cost per patient for the machine, annuitizing for a 10 year life span, is £32 per patient if you assume each site sees 300 patients per year. Alternatively, the per patient cost is £55 if you assume each site sees 200 patients per year.

If it is assumed that the procedure is carried out by a consultant, takes half an hour and there is a mixed model of care between nurses and health care assistants (half and half each) the total staff costs per patient are £64. If the procedure takes 45 minutes it is £97 per patient using the same mix of staff (overheads and oncosts are included in the salary costs).

As a result the total cost of an SLT is likely to be between £96 and £151 depending on the assumptions made. We have used the upper estimate of £151 as the cost per patient for an SLT to use the more conservative estimate.

Descriptive statistics for SLTs are reported in the main paper, Table 5. The average total cost per patient for SLT is reported in Table SM2.

4.3. Ocular related costs

Descriptive statistics for Medicine, surgery and IOP appointments are reported in the main paper (see Table 5). Total ophthalmology costs collected from patient files are reported in Table SM2. Patients randomised to Laser-1st had significantly higher costs for SLT and scheduled ophthalmology checks (excluding the two-week IOP check). For patients randomised to Medicine-1st they had significantly higher costs for Medicine, ocular surgery (including pre-operative assessment) and IOP checks. For all ophthalmology related costs, Medicine-1st cost an additional £451 (95% CI -£580 to -£322) unadjusted and an additional £447 (95% CI -£573 to -£322) for the adjusted analysis with bootstrapped bias corrected confidence intervals.

There were no significant differences between the two groups for community eye related costs collected using the CSRI (see Table SM3).

**Cost component**	**Medicine-1st**	**Laser-1st**	**Difference**
	n=362	n=356	
	Average (SD)	Average (SD)	Average (95% CI)
SLT	3 (22)	208 (82)	205 (196 to 213)
Medicine	526 (202)	61 (144)	-465 (-491 to -440)
Ocular Surgery	242 (709)	109 (386)	-134 (-218 to -50)
Surgery pre-operative assessment	17 (50)	8 (32)	-9 (-15 to -3)
Surgery post-operative assessment	1 (14)	0.3 (5)	-0.5 (-2 to 1)
IOP checks (excluding 2-week IOP check)	170 (290)	34 (111)	-135 (-168 to -103)
Scheduled Checks	446 (144)	535 (150)	90 (68 to 111)
Unscheduled Checks	26 (86)	21 (57)	-5 (-16 to 5)
3-year check	63 (42)	67 (40)	4 (-2 to 10)
**Total**	**1495 (1083)**	**1044 (608)**	**-451 (-580 to -322)**

Table SM2: Total average cost per patient of ophthalmology related appointments taken from patient files over 3 years (unadjusted). Costs in 2016/2017 GBP.

**Cost component**	**Medicine-1st**		**Laser-1st**		**Difference**
	Baseline	3-years	Baseline	3-years	
	n=354	n=223	n=348	n=217	
	Average (SD)	Average (SD)	Average (SD)	Average (SD)	Average (95% CI)
Optometrist	49 (37)	125 (95)	47 (39)	139 (111)	14 (-7 to 35)
Community costs* – eye related	7 (16)	23 (43)	7 (16)	19 (50)	-4 (-12 to 5)
Total	**56 (43)**	**133 (109)**	**54 (45)**	**141 (130)**	**8 (-14 to 30)**

* Community costs include GP, Primary Care nurse and social care

Table SM3: Eye related costs taken from completed CSRI over 3 years (discounted and unadjusted. Baseline values are 6 months prior to randomisation). Costs in 2016/2017 GBP.

4.4. Other health care resource use

There are no significant differences between the two arms for health care costs collected using the CSRI (see Table SM4) with a difference of -£319 (95% -£757 t0 118) for the adjusted analysis with 95% bias corrected bootstrapped confidence intervals. If missing data is imputed using chained equations the adjusted discounted difference is £36 (95% CI -£366 to £437).

Inpatient costs have been calculated separately given that information on inpatient stays could be supplemented with SAE data. The mean inpatient cost over 3 years (discounted) for patients randomised to Medicine-1st is £799 (SD 2592) with a mean cost of £1095 (3252) for the for patients randomised to Laser-1st, and an adjusted difference of £336 (95% CI -£97 to £770) with confidence intervals calculated from bias corrected bootstrap.

Cost component	Medicine-1st		Laser-1st		Difference
	Baseline	3-years	Baseline	3-years	
	n=354	n=224	n=348	n=231	
	Average (SD)	Average (SD)	Average (SD)	Average (SD)	Average (95% CI)
GP including Practice nurse	47 (65)	138 (171)	48 (79)	133 (133)	-5 (-36 to 26)
Social care	4 (40)	27 (109)	2 (15)	26 (134)	-1 (-25 to 22)
A&E Attendances	9 (40)	60 (163)	13 (51)	51 (147)	-9 (-39 to 22)
Acute outpatient (excludes eye related)	115 (182)	525 (683)	97 (172)	436 (706)	-89 (-227 to 49)
Day cases	256 (610)	1184 (2071)	230 (602)	920 (1577)	-264 (-619 to 90)
Total	**425 (712)**	**1776 (2538)**	**386 (719)**	**1389 (2100)**	**-387 (-815 to 41)**

Table SM4: Costs collected using the CSRI for non-eye related health care resource use over 3 years (discounted and unadjusted. Baseline values are 6 months prior to randomisation. Inpatient data includes SAE costs). Costs in 2016/2017 GBP.

4.5. Total health and social care costs

Total costs include the cost of SLTs, Medicine, eye related costs and non-eye related health and social care costs. Including all costs with no imputation the total adjusted cost for patients randomised to Medicine-1st over 3 years, discounted is £3993 (Standard Error (SE) 215 95% CI £3571 to £4414) and for Laser 1st it is £3890 (SE 245 95% CI £3409 to £4371) with a difference of -£103 (SE 325 95% CI -£739 to £534 p=0.752). Including imputed missing community data the difference in costs is -£105 (SE 348 95% CI -£788 to £579 p=0.764).

4.6. ICER and CEAC

For ophthalmology and total costs, Laser-1st dominates Medicine-1st in that it results in more QALYs for a lower cost. For ophthalmology only costs, the results of the MI (QALYs imputed only) and bootstrap, accounting for correlation between costs and QALYs using SUR, is that Laser results in an average cost saving of -£458 per patient with 95% of iterations falling between -£585 and -£345 and results in 0.014 additional QALYs with 95% bootstrap replications falling between -0.018 and 0.046. If non-eye related costs are also included the average cost saving per patient of Laser is -£126 with 95% of bootstrap replications falling between -£796 and £487.

The CEAC is presented in Figure 1. If ophthalmology costs only are included, at a £20,000 and £30,000 willingness to pay for a QALY gained, there is a 97% and 93% probability respectively that Laser-1st is cost-effective compared to Medicine-1st over 3 years, discounted and adjusted. For all health care related costs, including non-eye related costs, at both a £20,000 and £30,000 willingness to pay for a QALY gained there is a 68% chance that Laser-1st is cost-effective compared to Medicine-1st, discounted, adjusted and over 3 years.

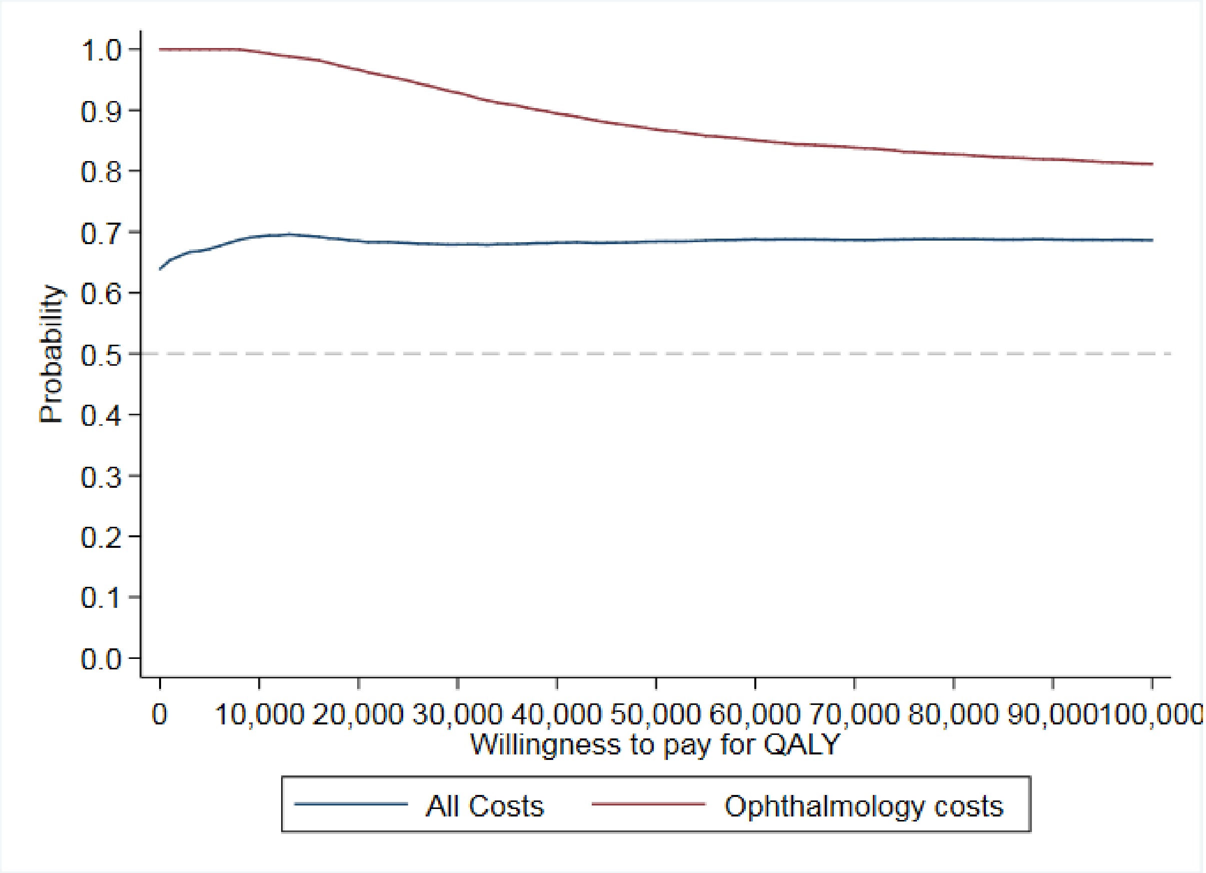

Figure SM1: CEAC of Laser-1st compared to Medicine-1st based on bootstrapped, imputed, discounted, adjusted data – Ophthalmology costs and all health care costs.

4.7. Sensitivity analysis

1) If SLT cost at the lower end of the micro-costing estimate, Laser-1st would result in £176 in cost savings compared to Medicine-1st for all health care costs. The cost of an SLT based on NHS Reference Costs is £188. If this value is used Laser-1st results in £89 in cost-savings if all health care costs are included.

2) The average cost of the two week following SLT check in the Laser arm was £128 and £20 in the Medicine-1st arm, with the two week check costing an additional £108 for patients randomised to Laser-1st. If the two week check is included in the analysis Laser-1st results in £18 of cost savings if all health care costs are included.

3) In the complete case analysis, the Laser-1st arm has a mean of 2.63 adjusted and discounted QALYs across 3 years (n=261 95% CI 2.60 to 2.65), with 2.61 QALYs in the Medicine-1st arm (n=263 95% CI 2.57 to 2.62) with and adjusted difference of 0.032 (95% CI -0.003 to 0.068 p=0.075). Laser-1st dominates Medicine-1st, with more costs and less QALYs.

5. References

Beecham JK, Knapp MRJ Costing psychiatric interventions. In: Thornicroft G, Brewin C, Wing JK (eds) Measuring Mental Health Needs. Gaskell: London; 1992. 200–224.

Curtis L, Burns A. Unit Costs of Health and Social Care 2017. *Personal Social Services Research Unit,* Kent, 2018.

Devlin NJ, Shah KK, Feng Y, Mulhern B, van Hout B. Valuing health-related quality of life: An EQ-5D-5L value set for England. *Health Economics*, 2017; **1-16.**

DH PbR Finance and Costing Team. National Schedule of Reference Costs 2016-17. Department of Health, London, 2018

Drummond MF, Sculpher MJ, Claxton K, Stoddart GL, Torrance GW. Methods for the Economic Evaluation of Health Care Programmes: Fourth Edition. Oxford University Press, Oxford, UK, 2015.

Georghiou T, Bardsley M. Exploring the cost of care at the end of life. Nuffield Trust, 2014.

Hunter RM, Baio G, Butt T, Morris S, Round J, Freemantle N. An Educational Review of the Statistical Issues in Analysing Utility Data for Cost-Utility Analysis. *PharmacoEconomics*, 2015; **33**(4): 355–66. doi: 10.1007/s40273-014-0247-6.

Joint Formulary Committee. British National Formulary (online) London: BMJ Group and Pharmaceutical Press <http://www.medicinescomplete.com>.

Leurent B, Gomes M, Faria R, Morris S, Grieve R, Carpenter J. Sensitivity Analysis for Not-at-Random Missing Data in Trial-Based Cost-Effectiveness Analysis: A Tutorial. *PharmacoEconomics*, 2018; 1-13 https://doi.org/10.1007/s40273-018-0650-5

National Institute for Health and Clinical Excellence. Guides to the methods of technology appraisal. 2013.

Violato M, Dakin H, Chakravarthy U, et al. Cost-effectiveness of community versus hospital eye service follow-up for patients with quiescent treated age-related macular degeneration alongside the ECHoES randomised trial. *BMJ Open* 2016;6:e011121. doi:10.1136/bmjopen-2016- 011121

Supplementary Online Material - 5

VF MD data.

	n	Laser-1st	n	Medicine-1st
**Baseline**		**Mean (SD) (dB)**		**Mean (SD) (dB)**
OHT	195	-1.51 (2.11)	185	-0.98 (1.95)
Mild POAG	309	-2.08 (1.85)	325	-2.16 (1.87)
Moderate POAG	67	-7.97 (2.05)	77	-8.08 (2.13)
Severe POAG	40	-9.58 (4.40)	35	-10.47 (4.65)
**12 months**				
OHT	189	-1.26 (2.40)	169	-1.06 (2.16)
Mild POAG	310	-2.21 (1.92)	330	-2.12 (1.94)
Moderate POAG	54	-7.20 (2.61)	56	-7.41 (2.23)
Severe POAG	44	-9.49 (4.55)	45	-9.52 (5.61)
**24 months**				
OHT	170	-1.01 (1.96)	150	-0.96 (2.08)
Mild POAG	276	-2.08 (1.93)	303	-2.25 (1.93)
Moderate POAG	68	-7.60 (2.68)	62	-7.97 (2.30)
Severe POAG	38	-10.52 (5.12)	36	-10.68 (4.60)
**36 months**				
OHT	152	-1.05 (1.98)	132	-0.94 (1.92)
Mild POAG	255	-1.99 (1.93)	254	-2.14 (1.95)
Moderate POAG	55	-7.96 (2.04)	66	-7.21 (1.92)
Severe POAG	52	-10.24 (4.93)	42	-10.50 (5.01)

Supplementary Online Material - 6

HRT neuroretinal rim area data.

	n	Laser-1st	n	Medicine-1st
**Baseline**		**Mean (SD) (mm^2)**		**Mean (SD) (mm^2)**
OHT	186	1.34 (0.34)	168	1.30 (0.33)
Mild POAG	284	1.10 (0.33)	300	1.10 (0.35)
Moderate POAG	56	1.03 (0.38)	70	1.04 (0.34)
Severe POAG	35	0.93 (0.31)	29	0.88 (0.31)
**12 months**				
OHT	183	1.35 (0.35)	163	1.27 (0.31)
Mild POAG	295	1.10 (0.34)	317	1.10 (0.33)
Moderate POAG	48	0.99 (0.45)	52	0.96 (0.32)
Severe POAG	42	0.94 (0.31)	40	0.94 (0.32)
**24 months**				
OHT	161	1.38 (0.35)	141	1.29 (0.32)
Mild POAG	253	1.09 (0.35)	282	1.07 (0.33)
Moderate POAG	58	1.06 (0.41)	55	0.99 (0.32)
Severe POAG	36	0.99 (0.34)	33	1.02 (0.45)
**36 months**				
OHT	140	1.31 (0.35)	124	1.28 (0.32)
Mild POAG	198	1.11 (0.36)	203	1.07 (0.33)
Moderate POAG	44	0.92 (0.35)	58	1.00 (0.30)
Severe POAG	39	0.98 (0.37)	28	0.94 (0.47)
